# Gut microbiota accelerates cisplatin-induced acute liver injury associated with robust inflammation and oxidative stress in mice

**DOI:** 10.1186/s12967-021-02814-5

**Published:** 2021-04-13

**Authors:** Shenhai Gong, Yinglin Feng, Yunong Zeng, Huanrui Zhang, Meiping Pan, Fangjie He, Rong Wu, Jingrui Chen, Jiuling Lu, Siyou Zhang, Songhua Yuan, Xia Chen

**Affiliations:** 1 Department of Obstetrics and Gynecology, First People’s Hospital of Foshan, Foshan, China; 2 School of Traditional Chinese Medicine, Southern Medical University, Guangzhou, China; 3 Department of Outpatient, First People’s Hospital of Foshan, Foshan, China

**Keywords:** Gut microbiota, Cisplatin, Hepatotoxicity, Inflammation, Oxidative stress

## Abstract

**Background:**

Gut microbiota has been reported to be disrupted by cisplatin, as well as to modulate chemotherapy toxicity. However, the precise role of intestinal microbiota in the pathogenesis of cisplatin hepatotoxicity remains unknown.

**Methods:**

We compared the composition and function of gut microbiota between mice treated with and without cisplatin using 16S rRNA gene sequencing and via metabolomic analysis. For understanding the causative relationship between gut dysbiosis and cisplatin hepatotoxicity, antibiotics were administered to deplete gut microbiota and faecal microbiota transplantation (FMT) was performed before cisplatin treatment.

**Results:**

16S rRNA gene sequencing and metabolomic analysis showed that cisplatin administration caused gut microbiota dysbiosis in mice. Gut microbiota ablation by antibiotic exposure protected against the hepatotoxicity induced by cisplatin. Interestingly, mice treated with antibiotics dampened the mitogen-activated protein kinase pathway activation and promoted nuclear factor erythroid 2-related factor 2 nuclear translocation, resulting in decreased levels of both inflammation and oxidative stress in the liver. FMT also confirmed the role of microbiota in individual susceptibility to cisplatin-induced hepatotoxicity.

**Conclusions:**

This study elucidated the mechanism by which gut microbiota mediates cisplatin hepatotoxicity through enhanced inflammatory response and oxidative stress. This knowledge may help develop novel therapeutic approaches that involve targeting the composition and metabolites of microbiota.

**Supplementary Information:**

The online version contains supplementary material available at 10.1186/s12967-021-02814-5.

## Background

Cisplatin, a first-generation platinum-containing drug, is effective in the treatment of various solid tumours, including lung, testicular, and ovarian tumours, as well as malignant pleural mesothelioma and breast cancer [1, 2]. The mechanism of cisplatin’s antitumor effect is attributed to DNA adduct formation, leading to interference with DNA synthesis and subsequent destruction of cancer cells during cell division [3, 4]. Mounting evidence suggests the existence of severe toxicity and undesirable adverse effects with the use of cisplatin [5]. Its toxicities mainly include ototoxicity, gastrotoxicity, nephrotoxicity, and hepatotoxicity, all of which severely limit clinical application [6, 7]. Drug-induced liver injury is a significant health problem because of its unpredictable nature and possible fatal outcome [8]. Patients with acute liver failure normally have poor prognoses and quality of life. Recently, the hepatotoxicity in patients receiving high-dose cisplatin treatment has gained widespread attention; unfortunately, the molecular mechanisms responsible for the toxicity are not clearly understood, resulting in the lack of a proper treatment to prevent liver injury induced by cisplatin during clinical usage [5, 9, 10]. The current study was designed to explore the pathogenesis of cisplatin-induced liver failure and provide potential treatment strategies.

Gut microbiota and metabolites have emerged as key factors in modulating the pathological processes of liver disease [11, 12]. Our previous work has shown that the microbial metabolites 1-phenyl-1, 2-propanedione could directly decrease hepatic glutathione and enhance oxidative stress, thereby predisposing mice to acetaminophen hepatotoxicity [13]. Recent observations have highlighted that gut microbiota-derived metabolites could translocate to the liver through the leaky gut, subsequently aggravating the host inflammatory response by binding TLR4 receptors and activating the mitogen-activated protein kinase (MAPK) pathway [14]. In addition, gut microbiota has been reported to modulate the expression of key hepatic cytochrome enzymes, influencing drug metabolism [15, 16]. There are growing evidence that the gut microbiota modulates host response to the efficacy and toxicity of chemotherapeutic drugs [17, 18]. The interaction between cisplatin hepatotoxicity and intestinal microbiota requires exploration, as the detailed mechanism will assist in determining a suitable clinical approach. In the present study, we described a new mechanism through which gut microbiota significantly promote cisplatin hepatotoxicity by enhancing the host inflammatory response and oxidative stress, in order to shed light on the potential therapeutic strategies against cisplatin-induced liver injury by targeting the gut microbiota.

## Materials and methods

### Animal model

Male specific-pathogen-free C57BL/6 mice (6 to 8 weeks old) were injected intraperitoneally with 30 mg/kg cisplatin dissolved in phosphate-buffered saline (PBS) and were sacrificed 24 h after cisplatin treatment. Faecal microbiota transplantation (FMT) was performed according to a method previously described [19]. Briefly, mice were administered antibiotics (vancomycin: 100 mg/kg; neomycin sulphate, metronidazole, and ampicillin: 200 mg/kg) intragastrically once daily for 5 consecutive days. The faecal supernatants of cisplatin and control donors were mixed and used as a single source for the cisplatin-FMT and control-FMT mice, respectively. Following antibiotic treatment, the recipient mice were orally inoculated daily for 3 consecutive days with prepared faecal contents. Three days after the first microbial administration, all mice were injected with 30 mg/kg cisplatin and sacrificed 24 h later. For antibiotic treatment, mice were gavaged with the antibiotics mentioned above for 3 consecutive days and then injected with 30 mg/kg cisplatin. Another group of male mice was administered as described above, but the antibiotics were replaced with PBS. All the mice were fed adlibitum and maintained in a temperature-controlled environment on a 12 h light–12 h dark cycle. All experimental procedures were in agreement with the National Institutes of Health guidelines and were approved by the local Animal Care and Use Committee of the Southern Medical University.

### Histological examination

Tissue was collected and fixed in 4% paraformaldehyde. The sample was then embedded (in paraffin), sliced, and stained with haematoxylin and eosin (H&E). The hepatic HE scores was assessed according to previous research [20, 21]. Briefly, the histological parameters of necrosis, inflammation, hepatocyte vacuolization were each accessed by using scale ranging from 0 to 3 (0 defined as absent; 3 defined as severe).

Terminal deoxynucleotidyl transferase dUTP nick end labelling (TUNEL) staining (KeyGene, Nanjing, China) was performed according to the manufacturer’s instructions. For apoptotic cell quantification, the percentage of positive cells having nuclear staining was calculated in 6–8 random fields per slide.

### Western blot analysis

Total protein was extracted using a commercial lysis buffer (Thermo Scientific, MA, USA). Primary antibodies targeting phospho-c-Jun N-terminal kinase (p-JNK; Cell Signaling Technology, MA, USA), JNK (Cell Signaling Technology, MA, USA), phospho-extracellular signal-regulated kinase (p-ERK; Cell Signaling Technology, MA, USA), ERK (Cell Signaling Technology, MA, USA), p-p38 (Cell Signaling Technology, MA, USA), p38 (Cell Signaling Technology, MA, USA), tumour necrosis factor alpha (TNF-α; Cell Signaling Technology, MA, USA), cytochrome P450 2E1 (CYP2E1; Proteintech, Wuhan, China), proliferating cell nuclear antigen (PCNA; Proteintech, Wuhan, China), glyceraldehyde-3-phosphate dehydrogenase (GAPDH; Cell Signaling Technology, MA, USA) and actin (Cell Signaling Technology, MA, USA) were used.

### Biochemical analysis

Serum alanine aminotransferase (ALT) and aspartate transaminase (AST) levels were measured using commercial kits (Jiancheng Bioengineering, Nanjing, China) according to the manufacturer’s instructions.

### Dihydroethidium staining

Dihydroethidium (DHE) (Invitrogen, MA, USA) was used to evaluate hepatocellular reactive oxygen species (ROS) levels in frozen sections of liver tissues. Sections of 6 μm thickness were washed thrice with PBS (PH 7.4) and then incubated with the fluorescent probe DHE (2 μM) at 37 °C for 30 min. Representative images were captured using a fluorescence microscope (Zeiss, Germany).

### Metabolomic analysis

A nontargeted metabolomics procedure was performed according to a previously described method via electrospray ionization quadrupole time-of-flight mass spectrometry (ESI-QTOF/MS) (Xevo G2-S Q-TOF, Waters, MA, USA) and ultra-high performance liquid chromatography-quadrupole time-of-flight mass spectrometry (UPLC-QTOF/MS) (ACQUITY UPLC I-Class, Waters, MA, USA) [22]. Samples were prepared and injected to an ACQUITY UPLC BEH C18 chromatographic column (130 Å, 1.7 µm, 2.1 mm × 50 mm, 1/pkg). Mobile phase A was 0.1% methane acid water and mobile phase B was 0.1% methane acid acetonitrile. The gradient elution was performed as follows: 0–2 min, 90% A and 10% B; 2–5 min, 70% A and 30% B; 5–8 min, 20% A and 80% B; 8–8.1 min, 10% A and 90% B; 8.1-10 min, 90% A and 10% B. The volume of the injected sample was 10 μL, and the flow rate was 0.4 mL/min. The capillary was set to 2 kV, and the cone voltages were set to 25 V. The acquisition rate was set to 0.2 s. The nebulisation gas was set to 800 L/h at a temperature of 500 °C, the cone gas was set to 50 L/h, and the source temperature was set to 120 °C.

### Microbial analysis

The mice were anaesthetized and faeces were collected, frozen immediately in liquid nitrogen, and stored at − 80 °C. The total DNA was extracted from faecal contents as previously described [23]. The faecal contents were resuspended separately in a PBS (pH 7.4) containing 0.5% Tween 20 and vortexed gently, followed by a − 80 °C/60 °C cycle three times to disrupt bacterial membranes. DNA extraction was performed using the phenol–chloroform method. The DNA extracted from the faecal contents was used to amplify the variable region 4 (V4) of the bacterial 16S rRNA gene using polymerase chain reaction (PCR). The V4-16S rRNA gene was amplified using barcoded primers (V4F, 5′-GTGTGYCAGCMGCCGCGGTAA-3′ and V4R, 5′-CCGGACTACNVGGGTWTCTAAT-3′), according to the manufacturer’s instructions for Illumina Hiseq PE250.

### Gene expression analysis

Total RNA was extracted from tissues using the TRIzol reagent according to the manufacturer’s instructions, and a reverse transcription enzyme (TOYOBO, Shanghai, China) was employed according to the manufacturer’s instructions. Real-time PCR was carried out on an ABI 7500 real-time PCR system. The real-time qPCR primers are shown in Additional file 1: Table S1.

### Glutathione assay

Reduced glutathione (GSH) was measured in liver homogenate using a reduced GSH assay kit (Jiancheng Bioengineering, Nanjing, China) according to the manufacturer’s instructions.

### Malondialdehyde (MDA) in liver

A Malondialdehyde (MDA) Detection Kit (Jiancheng Bioengineering, Nanjing, China) was selected to determine the MDA level as a marker of lipid peroxidation. The assay was conducted according to the manufacturer’s instructions.

### Statistical analysis

The results are expressed as the mean ± standard error of the mean (SEM). A two-tailed Student’s t-test was used for statistical evaluation. Multiple hypotheses were adjusted using the Benjamini and Hochberg method. Statistical differences between groups were considered significant at *p* < 0.05. We clustered and annotated the 16S rRNA gene reads into amplicon sequence variants (ASVs) using the DADA2 pipeline and Greengenes Database V.13_8 in QIIME2 [24, 25]. Values for alpha diversity [Chao1 index, Shannon’s index, phylogenetic diversity (PD) whole tree index, and observed operational taxonomic units (OTUs)], beta diversity (Binary Jaccard distance metrics), and principal coordinate analysis (PCoA) were generated using QIIME2. Linear discriminant analysis effect size (LEfSe) was performed to determine the features most likely to explain the differences between groups [26]. Phylogenetic Investigation of Communities by Reconstruction of Unobserved States 2 (PICRUSt2) analysis based on KEGG pathway was performed to infer the microbial related pathways [27]. Principal component analysis (PCA) was performed for metabolomic data using the R package vegan. The R package DESeq was applied to determine the variation tendency of metabolites between groups. Spearman’s rho correlation test was performed for network correlation and P values were adjusted by the Benjamini and Hochberg method. Metabolomic pathway enrichment analysis was performed using the online tool MetaboAnalyst [28]. Heatmap, volcano plot, and bubble plot were plotted using R package pheatmap, Enhanced Volcano, and ggplot2, respectively.

## Result

### Cisplatin treatment induced liver injury in mice

**Fig. 1:**
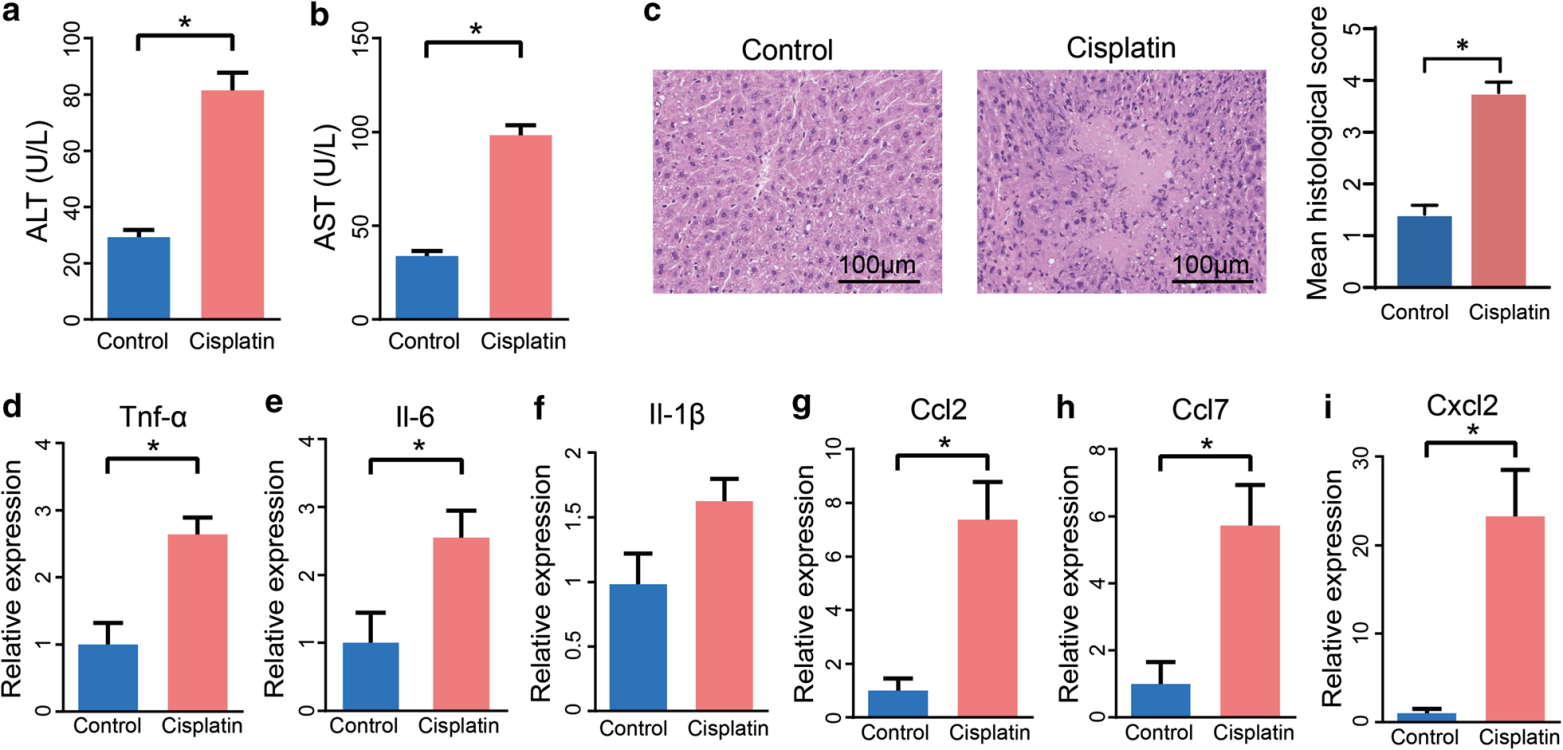
Cisplatin induced liver injury in mice. **a**, **b** Plasma ALT and AST levels after 24 h cisplatin treatment. **c** H&E staining and histological score of liver tissue after 24 h cisplatin treatment. **d**–**i** mRNA levels of key cytokines and chemokines in the liver. The results are expressed as mean ± SEM. n = 4–10 per group. *P < 0.05. *ALT* alanine aminotransferase, *AST* aspartate aminotransferase, *H&E* Hematoxylin and Eosin

First, mice were intraperitoneally administered a single dose of cisplatin and the hepatotoxicity was detected 24 h later. As illustrated in Fig. 1a, b, cisplatin treatment significantly increased ALT and AST levels in plasma. The hepatic H&E staining indicated that mice also displayed more severe liver damage, mainly including necrosis, inflammation, hepatocyte vacuolization, upon cisplatin treatment (Fig. 1c). We detected that the levels of key cytokines and chemokines, such as TNF-α, IL-6, and IL-1β, were elevated in the liver after cisplatin injection (Fig. 1d–i). These results indicated that overdose of cisplatin induced hepatotoxicity in mice.

### Cisplatin induced gut microbiota dysbiosis at compositional level in mice

**Fig. 2:**
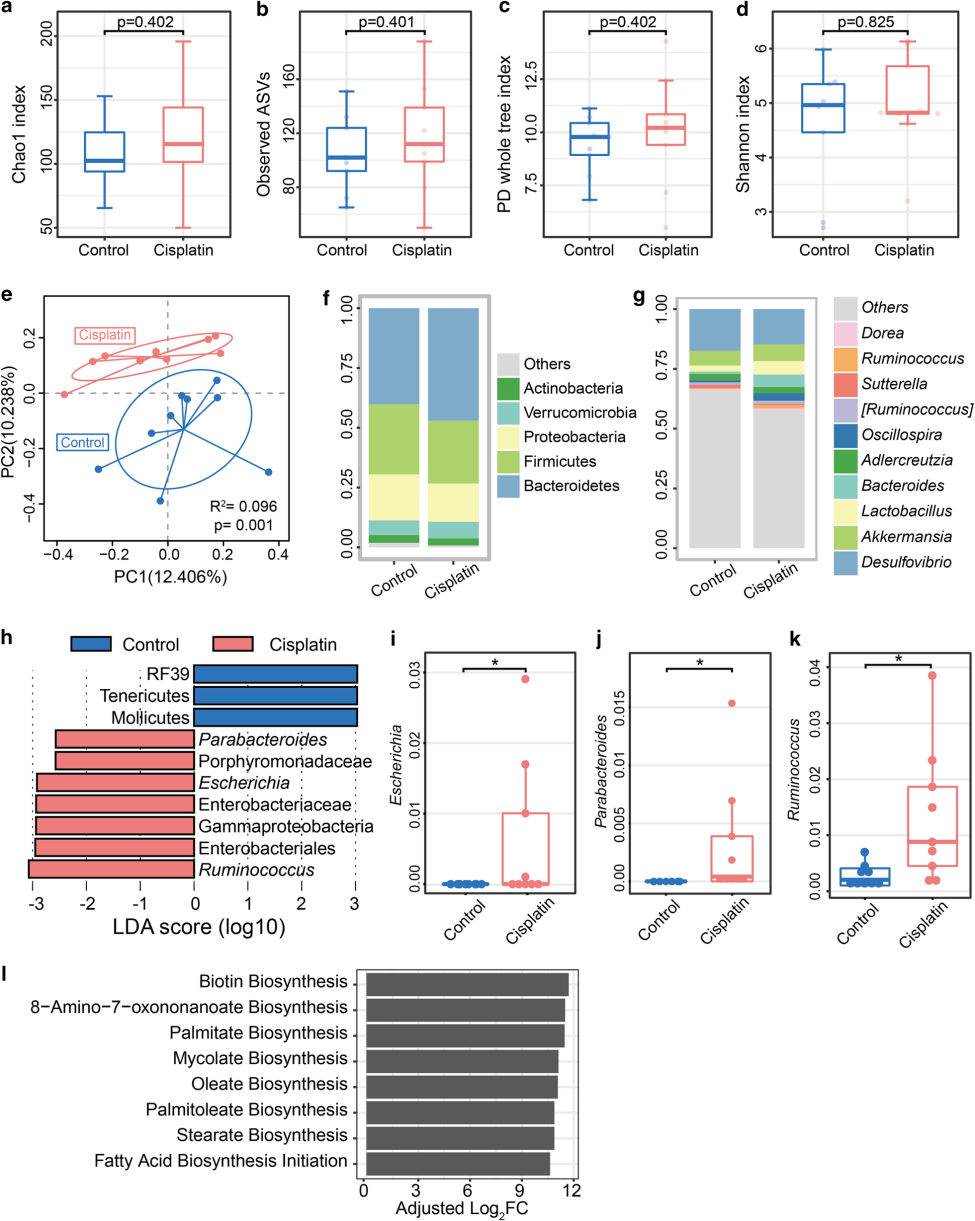
Cisplatin influenced the composition of faecal microbiota in mice. **a**–**d** Alpha diversity indices (Chao 1, observed OTUs, PD whole tree, and Shannon) in bacterial microbiomes. **e** Principal coordinate analysis (PCoA) based on the Binary Jaccard distance analysis of operational taxonomic units (OTUs). **f**, **g** Relative abundance at the phylum and genus level between control and cisplatin-treated groups. **h** LDA along with effect size measurements was applied to present the enriched bacteria in each group in faecal content. **i**–**k** Relative abundance of *Escherichia*, *Parabacteroides* and *Ruminococcus* between control and cisplatin-treated groups. **l** Predicted pathways enriched in cisplatin group as assessed by PICRUSt. n = 9 per group. *P < 0.05

We performed 16S rRNA gene sequencing to investigate whether cisplatin treatment alters the composition of gut microbiota in mice. As shown in Fig. 2a–d, there were no significant changes in the alpha-diversity of bacteria in terms of Chao1 index, Shannon’s index, phylogenetic diversity (PD) whole tree index, and observed OTUs in cisplatin-treated group compared to the control group. However, the PCoA of the Binary Jaccard distance showed a clear separation between the cisplatin and control microbial communities (Fig. 2e). In particular, we found that the abundance of *Bacteroidetes* was higher in the cisplatin group than in the control group at the phylum level. Moreover, at the genus level, an increased abundance of *Ruminococcus* was observed in the faeces of cisplatin-treated group compared to that in the faeces of control group (Fig. 2f, g). Additionally, according to the linear discriminant analysis along with the effect size measurement, we found that the specific bacterial linages were distinguishable in cisplatin-treated mice (Fig. 2h). For example, the relative abundance of *Escherichia* and *Parabacteroides* were enriched in the cisplatin-treated mice (Fig. 2i–k). We also performed PICRUSt analysis to predict the function of gut microbiota after cisplatin treatment in mice. As shown in Fig. 2l, we found several pathways enriched in cisplatin group, mainly including the biosynthesis of palmitoleate, palmitate, stearate and oleate. Collectively, these results demonstrated that cisplatin administration alters the composition of gut microbiota in mice.

### Cisplatin treatment changed gut microbial physiological function

**Fig. 3:**
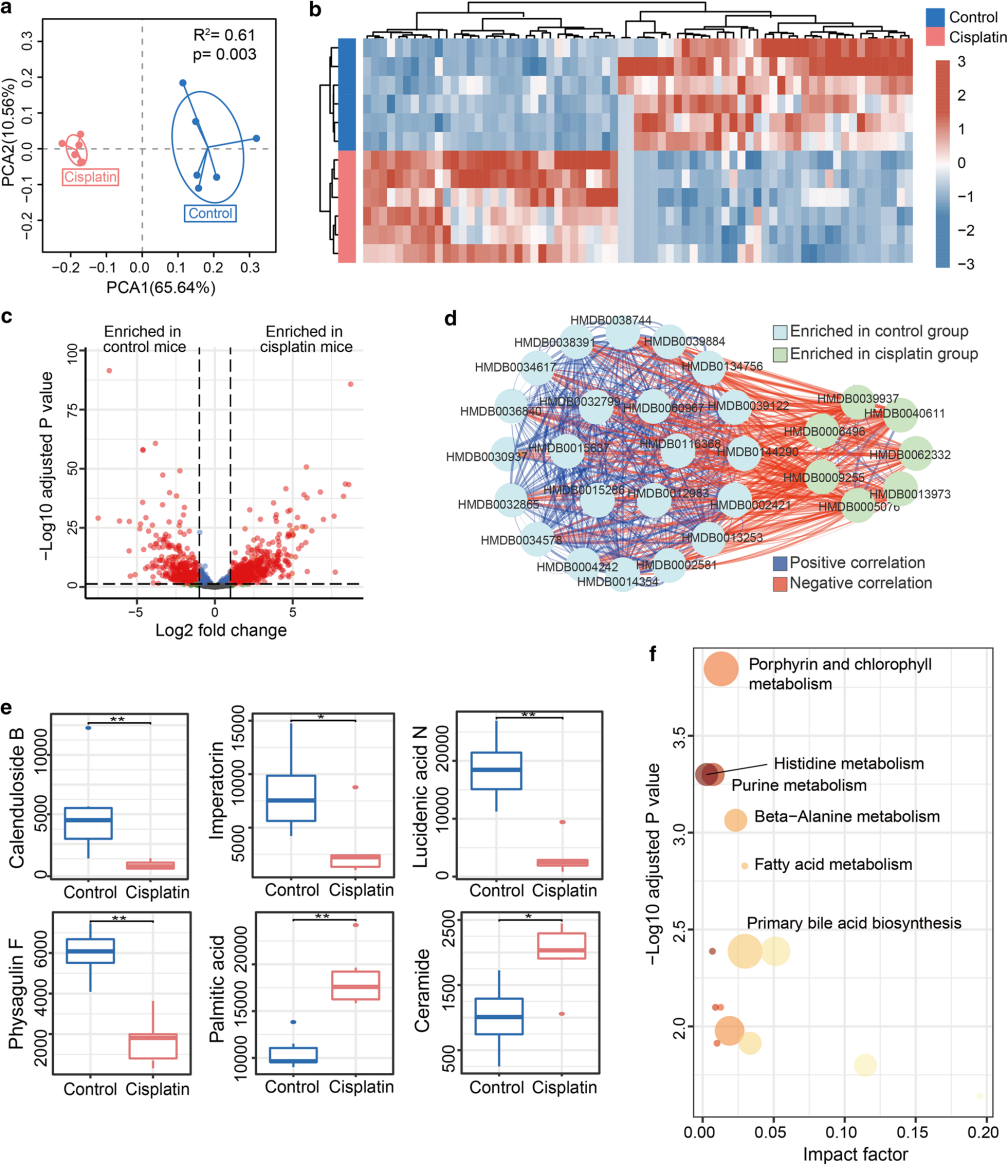
Cisplatin altered the microbiota metabolites. **a** Principal Component Analysis (PCA) analysis of the metabolomics of faecal content. **b** Heat map shows the differential expression of metabolites between faecal content from the control and cisplatin-treated group. **c** Volcano plot indicates the increase in differentially expressed metabolites between the groups. **d** Network analysis indicates the correlation within and between metabolites up-regulated in both groups. **e** Comparison of Calenduloside B, Imperatorin, Lucidenic acid N, Physagulin F, Palmitic acid, and Ceramide between Control and Cisplatin groups. **f** Functional annotation and enrichment analysis of certain important differential metabolites. *P < 0.05, **P < 0.01, n = 5 per group

Metabolomic analysis was performed to determine the difference in metabolites and functions in the cisplatin treatment group compared to control group. As shown in Fig. 3a, the PCA analysis showed a significantly shifted metabolic profile in cisplatin-treated mice. The heatmap also showed alteration of metabolites between the groups (Fig. 3b). The volcano plot represented the enrichment of differentially expressed metabolites in the two groups (Fig. 3c). The metabolites enriched in cisplatin group were positively correlated with each other but negatively associated with those enriched in control group (Fig. 3d). As shown in Fig. 3e, we found that level of several potential metabolites which could cause cell apoptosis and inflammatory response were enriched after cisplatin treatment [29, 30]. In particular, palmitic acid that could lead to insulin resistance by binding directly with MD2 to activate TLR4/NF-κB signal pathway [29]. Meanwhile, several potential metabolites which possess anti-inflammatory and anti-oxidation stress activities were reduced in cisplatin compared to control group [31–35]. It is reported that imperatorin could attenuate dextran sulphate sodium induced colitis by targeting pregnane X receptor to inhibit inflammatory response in mice [34]. In addition, prominent research efforts have elaborated that imperatorin could protect against drug induced liver injury associated with decrease level of inflammation cytokines and oxidative stress in mice [35]. We mapped metabolites to physiological pathways and observed significant differences in the levels of several pathways responsible for metabolism. In particular, metabolites involved in porphyrin and chlorophyll metabolism, histidine metabolism, and purine metabolism were significantly enhanced in faeces from cisplatin-treated group compared to that in faeces from the control group (Fig. 3f). Together, all results suggest that cisplatin treatment induced dysbiosis of gut microbial physiological function.

### Cisplatin hepatotoxicity was associated with gut microbiota dysbiosis

**Fig. 4:**
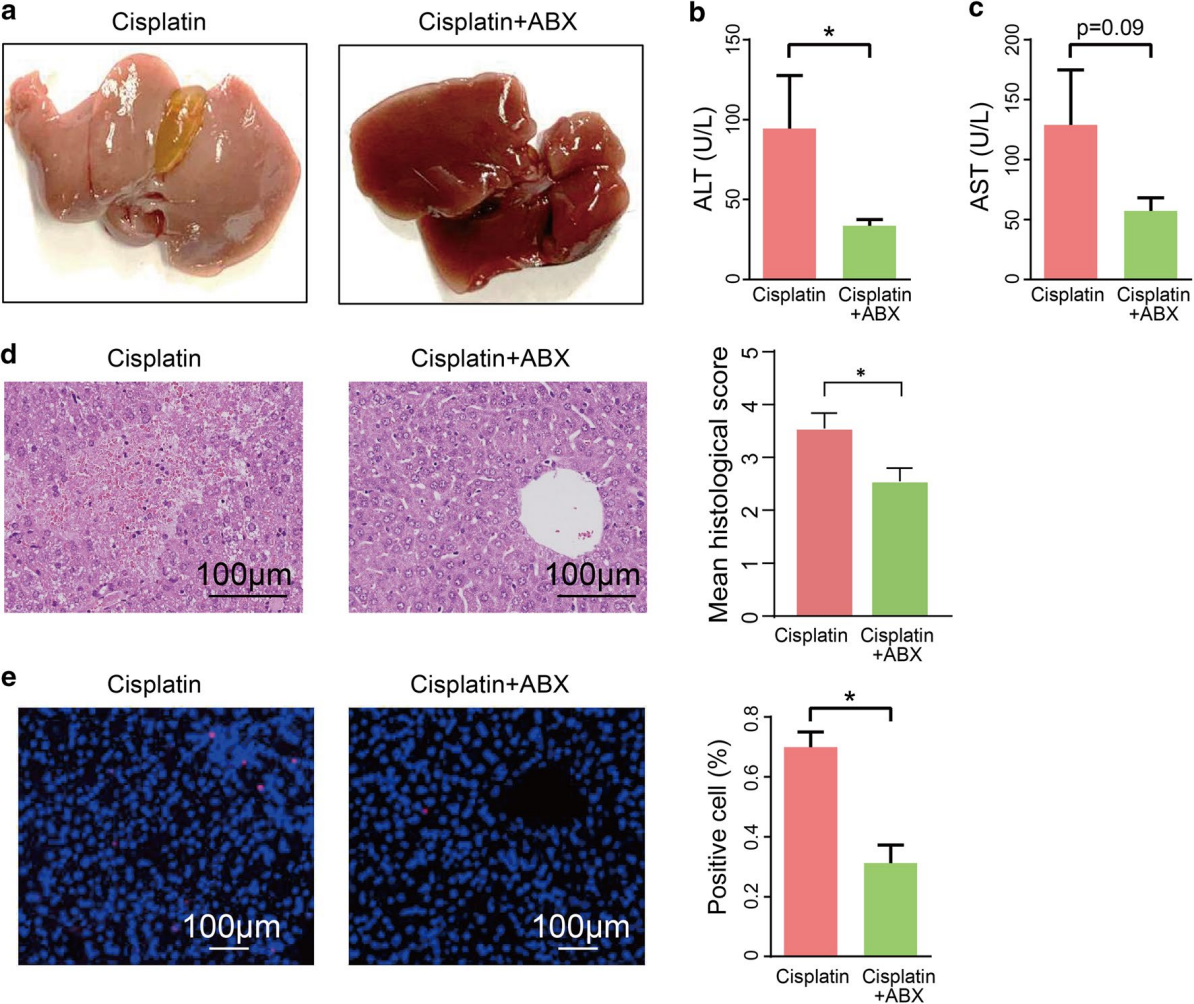
Gut microbiota mediated the hepatotoxicity induced by cisplatin. **a** Representative liver morphology. **b**, **c** Plasma ALT and AST levels after 24 h cisplatin treatment. **d** H&E staining and histological score of liver tissue after 24 h cisplatin treatment. **e** Hepatic TUNEL staining and quantification of liver. The results are expressed as mean ± SEM. n = 5–12 per group. *P < 0.05. *ALT* alanine aminotransferase, *AST* aspartate aminotransferase, *H&E* Hematoxylin and Eosin, *TUNEL* terminal deoxynucleotidyl transferase dUTP nick end labelling

Multi-omics results revealed that dysbiosis of the gut microbiota accompanied cisplatin-induced liver failure. We hypothesized that the dysbiosis of certain microbiomes or its derived metabolites would promote progression of cisplatin hepatotoxicity. To decipher the mechanisms, an antibiotic cocktail was used to deplete gut microbiota before cisplatin treatment; this significantly ameliorated cisplatin-induced liver injury as shown in Fig. 4a. The plasma ALT and AST levels were decreased in the ABX group compared to those in the control group (Fig. 4b, c). The hepatic HE scores also indicated that ABX treatment could decrease liver damage (Fig. 4d). Additionally, the protective effects of ABX were supported by the results of the liver histology TUNEL assay (Fig. 4e). Together, our data revealed that cisplatin-associated hepatic injury was suppressed in ABX-treated mice. Hence, we concluded that cisplatin-induced liver injury is mediated by gut microbiota.

### Antibiotic treatment alleviated cisplatin induced oxidative stress and inflammation in liver

**Fig. 5:**
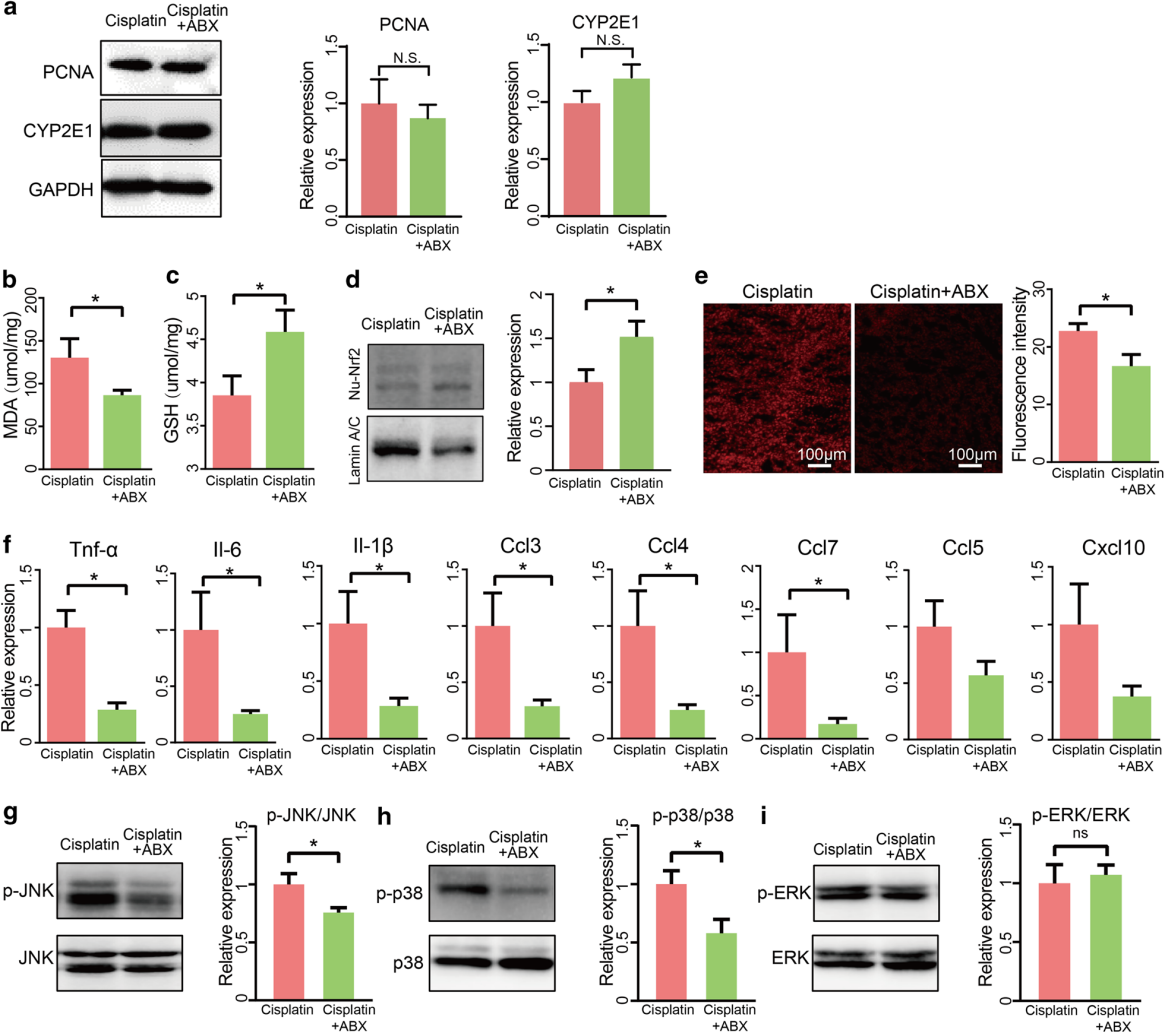
Oxidative stress and inflammation were suppressed after cisplatin treatment in ABX group. **a** Western blot and quantification of PCNA and CYP2E1 in liver tissue. **b** Malondialdehyde (MDA) levels in mice liver. **c** Glutathione (GSH) levels in the liver homogenates after cisplatin treatment for 24 h. **d** Western blot and quantification of Nrf2 in the liver in cisplatin and cisplatin + ABX group after cisplatin treatment for 24 h. **e** Representative image and quantification of dihydroethidium (DHE) immunofluorescence staining in cisplatin and cisplatin + ABX group after cisplatin treatment for 24 h. **f** mRNA levels of key cytokines and chemokines in the liver. **g**–**i** Western blot and quantification of p-JNK, p-p38, p-ERK in cisplatin and cisplatin + ABX group after cisplatin treatment for 24 h. The results are expressed as mean ± SEM. n = 3–12 per group. *P < 0.05

To examine the underlying mechanism by which gut microbiota enhances cisplatin hepatotoxicity, we first detected hepatic expression of CYP2E1 in cisplatin with or without antibiotic. Regretfully, we found that there is similar expression level in two groups (Fig. 5a). Additionally, hepatic PCNA also showed similar expression level in antibiotic-treated compared control group (Fig. 5a). It is generally accepted that the molecular mechanisms underlying cisplatin-induced liver injury are involved in oxidative stress and inflammatory response. To explore whether ABX treatment would affect hepatic oxidative stress and inflammatory response, we first detected that MDA levels, which were found to be decreased in the livers of the ABX group (Fig. 5b). We then compared the intracellular levels of GSH and reactive oxygen species (ROS) between the two groups and found that hepatic GSH concentrations were much higher in the ABX group than in the control group in the presence of cisplatin (Fig. 5c). Moreover, western blotting showed that ABX treatment promoted nuclear translocation of hepatic nuclear factor erythroid 2-related factor 2 (Nrf2) in mice, as shown in Fig. 5d. Compared to the control group, ABX treatment exhibited a lower accumulation of ROS, which is an indicator of oxidative stress in mice (Fig. 5e). Meanwhile, the majority of inflammatory factors were diminished after depletion of microbiota in the presence of cisplatin (Fig. 5f). We examined MAPK signalling pathways, deemed as regulators of inflammatory response, to further understand the mechanism that mediates the protective role of antibiotics. Our results showed that the phosphorylation of proteins involved in the JNK and p38 pathways was suppressed in the ABX treatment compared to that in the control group, whereas the phosphorylation of proteins involved in the ERK pathways was not significantly changed (Fig. 5g–i). Together, these results indicated that the absence of microbiota by ABX treatment reduced the inflammatory response and oxidative stress by inhibiting the phosphorylation of proteins involved in the JNK and p38 pathways and promoting the nuclear translocation of Nrf2 in cisplatin-treated mice.

### Gut microbiota from cisplatin treated mice augmented alone cisplatin hepatotoxicity

**Fig. 6:**
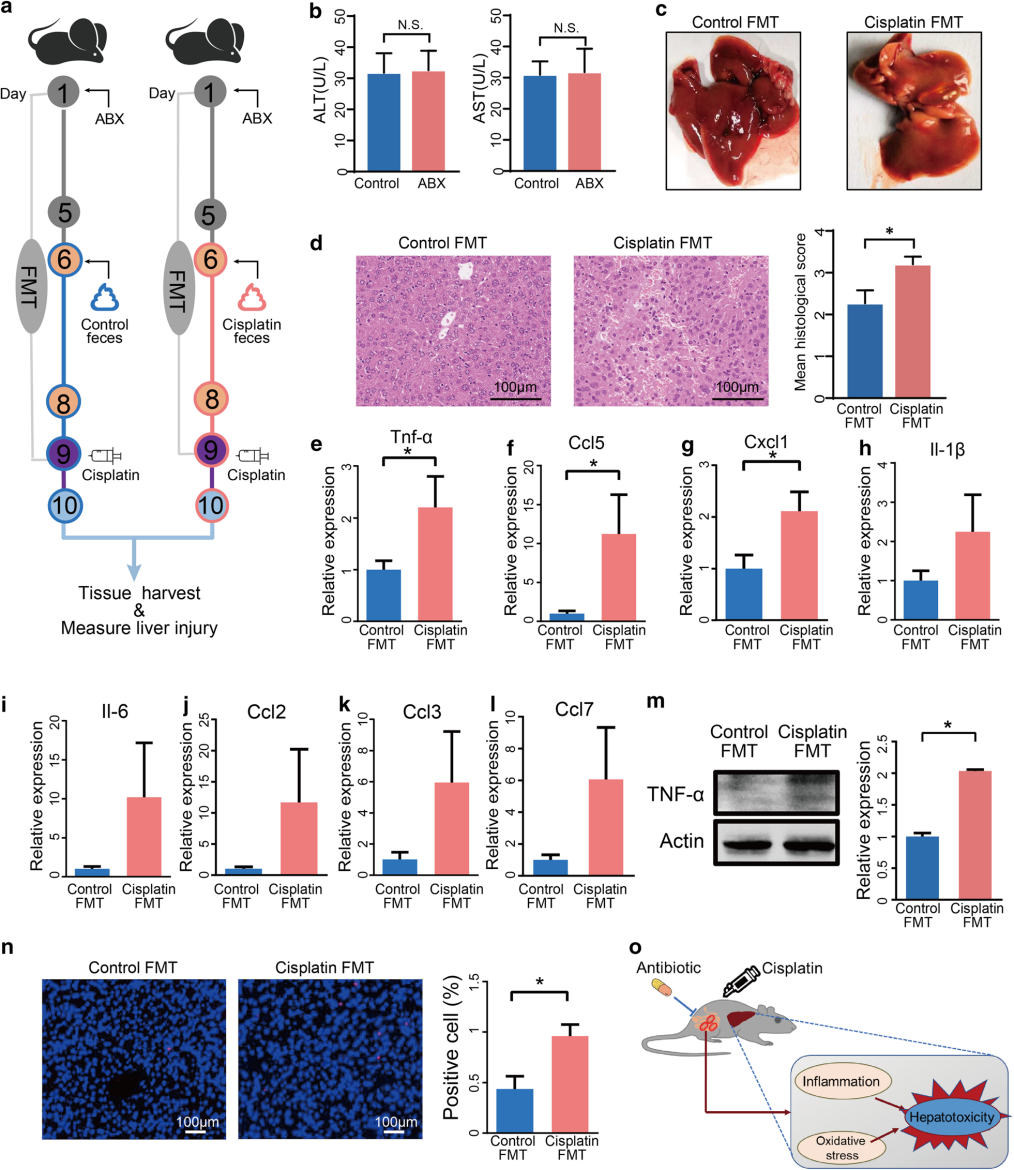
Faecal transplantation from cisplatin-treated mice augmented cisplatin hepatotoxicity. **a** FMT experimental design. **b** Plasma ALT and AST levels after antibiotics treatment. **c** Representative liver morphology. **d** H&E staining and histological score of liver tissue after 24 h cisplatin treatment. **e**–**l** mRNA levels of key cytokines and chemokines in the liver. **m** Western blotting and quantification of TNF-α level of liver of recipient mice. **n** Hepatic TUNEL staining and quantification of recipient mice. The results are expressed as mean ± SEM. n = 5–8 per group. *P < 0.05. **o** Working model: Gut microbiota accelerates cisplatin-induced hepatotoxicity through enhancing inflammation and oxidative stress pathways, which can be alleviated by antibiotics treatment. *FMT* faecal microbiota transplantation, *TUNEL* terminal deoxynucleotidyl transferase dUTP nick end labelling

To facilitate certification our hypothesis that cisplatin-associated gut microbiota alteration promotes cisplatin hepatotoxicity, an FMT experiment was performed. First, mice received vancomycin (100 mg/kg), neomycin sulphate (200 mg/kg), metronidazole (200 mg/kg), and ampicillin (200 mg/kg) once daily for 5 days to deplete gut microbiota, after which they received faeces resuspended in PBS from control or cisplatin-treated mice for 3 days (Fig. 6a). First, antibiotics administration alone as dose of once daily for 5 days did not cause hepatotoxicity in mice (Fig. 6b). The mice that received faeces of cisplatin-treated group exhibited more liver damage than the mice that received faeces from the control group after cisplatin treatment, as indicated by liver pathology (Fig. 6c, d). Next, we observed that certain key inflammatory factors, specifically TNF-α, Ccl5, and Cxcl1, were elevated in mice that received faeces from the cisplatin-treated group compared to the mice that received faeces from the control group (Fig. 6e–l). Increased hepatic TNF-α protein level also confirmed the mRNA expression levels in the liver (Fig. 6m). After FMT, the mice that received faeces from the cisplatin-treated group also showed more apoptosis, as revealed by TUNEL staining (Fig. 6n). These results indicated that the dysbiosis of gut microbiome induced by cisplatin treatment could promote the progression of cisplatin-induced liver failure.

## Discussion

Cisplatin has been effectively utilised as a powerful chemotherapeutic agent against many malignancies [36]. Previous preclinical studies have reported that cisplatin is known to induce severe liver damage, such as degeneration of hepatocytes [37, 38], along with significant renal toxicity [39]. Indeed, our results proved hepatotoxicity occurrence after cisplatin administration, indicated by elevated plasma level of ALT and AST in mice. However, the aetiology of cisplatin hepatotoxicity has not been clearly demonstrated. This study was conducted to investigate the potential role of gut microbiota in hepatotoxicity induced by cisplatin.

Emerging evidence has indicated that cisplatin could result in the occurrence of an imbalanced gut microbiota and decreased ecological diversity [40, 41]. Although alpha-diversity indices, including Shanno index and PD whole tree index, were not difference between two groups, our data indicated that cisplatin stimulation led to shift composition of gut microbiota, especially increased abundance of *Escherichia*, *Parabacteroides*, and *Ruminococcus*. Interestingly, most of these dysbiosis microbiota, belonging to gram-negative bacteria groups that are rich in LPS, may target pattern recognition receptors and initiate damage-associated molecular patterns [42, 43].

Apart from the compositional changes in gut microbiota, metabolomic analysis showed that the metabolites derived from intestinal microbiota were distinguishable in cisplatin stimulation compared to control group. Specifically, the level of imperatorin, physagulin F, lucidenic acid N and calenduloside B which were reported to promote health by modulating inflammation and oxidative stress were decreased in cisplatin group. However, cisplatin stimulation could increase ceramide, well defined as danger signal molecular to promoting development of disease. Interestingly, PICRUSt and metabolomic analysis both showed cisplatin treatment elevated level of palmitic acid. Taking these factors into account, we hypothesised that cisplatin-induced gut microbiota and dysbiosis of the derived metabolites may contribute to hepatotoxicity induced by cisplatin. To explore the precise role of microbiota in cisplatin-induced liver failure pathogenesis, we observed that mice treated with antibiotics ameliorated cisplatin-induced liver failure compared to normal mice based on results of plasma ALT and hepatic apoptosis. Most importantly, our FMT provided evidence that faeces from cisplatin-treated mice could augment cisplatin hepatotoxicity. This study provides insight that targeting microbiota treatment may be beneficial to attenuate liver damage induced by cisplatin.

Although the mechanism of cisplatin hepatotoxicity is not clearly understood, hepatic inflammatory response and oxidative stress seem to play a substantial role [20, 44]. We proposed that the gut microbiota or their derived metabolites may participate in oxidative stress and inflammation progression. Our findings confirmed that cisplatin hepatotoxicity was prevented by antibiotic treatment, allowing for improved liver function. In particular, our study also clearly indicated that antibiotic treatment could markedly promote Nrf2 activation and the level of glutathione, which maintains hepatic redox homoeostasis, is higher after cisplatin administration in ABX compared to that in the control group. Moreover, pre-treatment with antibiotics effectively inhibited JNK and p38 pathway activation and improved activation of cisplatin-induced hepatic inflammatory response in mice.

Antibiotic administration may alter the pharmacokinetics of cisplatin via drug-metabolizing enzymes and transporters. The gut microbiota has been reported to influence drug metabolism, consequently influencing drug efficacy and toxicity [45]. Previous studies also reported that the intestinal flora changes affect the expression of cytochrome P450 (CYP), which is one of the factors responsible for individual differences in pharmacokinetics [46]. Unfortunately, we found that there is similar expression level in two groups. It is reported that the mRNA level of CYP2E1 is not distinguished in germ free compared to conventional mice at 6–8 weeks old [47]. Therefore, we believed that gut microbiota enhanced hepatotoxicity of cisplatin and it may not be associated with CYP2E1.

Until now, effective treatments to protect against cisplatin hepatotoxicity have been limited in clinical practise. Further clinical research is necessary to reveal novel therapeutic approaches by targeting gut microbiota for prevention of cisplatin hepatotoxicity.

## Conclusions

In conclusion, our work established the precise role of gut microbiota in the regulation of cisplatin-induced liver failure by enhancing the hepatic inflammatory response and oxidative stress. These results could lead to the discovery of potential therapeutic strategies for the prevention of cisplatin hepatotoxicity.

## Supplementary Information

**Additional file 1: Table S1.** Primers used in quantitative-PCR analysis.

## Data Availability

The data are included in the article as figures, tables, and others which can email to the corresponding author.

## Abbreviations

FMT: Faecal microbiota transplantation
ABX: Antibiotics
H&E: Hematoxylin and Eosin
ALT: Alanine aminotransferase
AST: Aspartate aminotransferase
Ccl: Chemokine (C-C motif) ligand
Cxcl: Chemokine (C-X-C motif) ligand
Il: Interleukin
MAPK: Mitogen-activated protein kinase
p-ERK: Phosphorylated extracellular signal-regulated kinase
p-JNK: Phosphorylated c-Jun N-terminal kinase
p-p38: Phosphorylated p38
Tnf-α: Tumor necrosis factor alpha
Nrf2: Nuclear factor erythroid 2-related factor 2
TUNEL: Terminal deoxynucleotidyl transferase dUTP nick end labelling
ROS: Reactive oxygen species
GSH: Glutathione
MDA: Malondialdehyde
OUT: Observed operational taxonomic unit
PCoA: Principal coordinate analysis
LEfSe: Linear discriminant analysis effect size
PCA: Principal component analysis
PCNA: Proliferating cell nuclear antigen
CYP2E1: Cytochrome P450 2E1
GAPDH: Glyceraldehyde-3-phosphate dehydrogenase
